# Plasma omega-3 polyunsaturated fatty acids and recurrence of endometrial cancer

**DOI:** 10.1186/s12885-020-07035-5

**Published:** 2020-06-20

**Authors:** Peiqin Li, Boer Shan, Keyu Jia, Fan Hu, Ying Xiao, Jusheng Zheng, Yu-Tang Gao, Huaying Wang, Ying Gao

**Affiliations:** 1 CAS Key Laboratory of Nutrition, Metabolism and Food Safety, Shanghai Institute of Nutrition and Health, University of Chinese Academy of Sciences, Chinese Academy of Sciences, Shanghai, People’s Republic of China; 2 Department of Gynecologic Oncology, Fudan University Shanghai Cancer Center; Department of Oncology, Shanghai Medical College, Fudan University, Shanghai, China; 3 Macau Institute for Applied Research in Medicine and Health, Macau University of Science and Technology, Macau, China; 4 School of Life Sciences, Westlake University, Hangzhou, China; 5 Department of Epidemiology, Shanghai Cancer Institute, Shanghai, China

**Keywords:** Endometrial cancer, Omega-3 PUFAs, EPA, Recurrence

## Abstract

**Background:**

Omega-3 polyunsaturated fatty acids (PUFAs) were proposed to have potential effects against inflammation and cancer. However, results from epidemiology studies remain inconsistent. We aimed to explore the associations of plasma PUFAs with EC recurrence and all-cause mortality.

**Method:**

Women diagnosed with endometrial cancer (EC) between 2008 and 2013 and underwent surgery at Fudan University Shanghai Cancer Center of China were recruited. Survival status was followed up through September 2017. EC recurrence and total cause deaths were identified through medical record and telephone interview. In total, 202 patients with enough plasma samples at time of surgery were included. There were 195 patients who provided baseline plasma and survival information included in the current study. Plasma omega-3 PUFAs were measured by GC-FID. Cox Proportional Hazard model adjusted for potential cofounders was used to estimate HRs and 95% CIs.

**Results:**

Median follow-up time for patients was 58 months after surgery. A total of 13 recurrences and 11 all-cause deaths, of which, 2 deaths from EC, were identified. Level of plasma EPA was higher in recurrent patients than total patients (0.78% vs 0.51%, *P* = 0.015). Higher plasma eicosapentaenoic acid (EPA) level trended to have positive association with EC recurrence (*P*-trend = 0.04), although comparing to the lowest tertile, the highest tertile of EPA level was not significantly associated with increased risk of EC recurrence (HR_T3vsT1_ = 6.02; 95%CI = 0.7–52.06). The association between total omega-3 PUFA and EC recurrence tended to be stronger among patients with deeper myometrial invasion (OR = 3.41; 95%CI = 1.06–10.95; *P*-interaction = 0.04).

**Conclusions:**

Higher plasma EPA level was significantly associated with EC recurrence. Further studies are warranted to confirm these findings.

**Trial registration:**

ChiCTR1900025418; Retrospectively registered (26 August 2019); Chinses Clinical Trial Registry.

## Background

Endometrial cancer (EC) is the fifth most common cancer in women worldwide [1]. The incidence has increased 21% since 2008, and the mortality has grown more than 100% over the past 20 years [2]. Five-year survival rate of EC was about 90% for early stage, 57–66% for stage III, 20–26% for stage IV [3]. Therefore, early diagnosis and treatment are very important for EC prognosis. A meta-analysis showed that metabolic syndrome increased EC risk by 61% [4]. Fat accumulation which increases body fatness and promotes the development of obesity or metabolic syndrome may increase EC risk [5]. Understanding the role of lifestyle and nutrition factors may improve survival outcomes.

Omega-3 polyunsaturated fatty acids (PUFAs), including α-linolenic acid (ALA), eicosapentaenoic acid (EPA), docosapentaenoic acid (DPA), and docosahexaenoic acid (DHA), have been reported to slow down the growth of cancer cells and reduce tumor angiogenesis [6, 7]. Studies showed that dietary omega-3 PUFAs were associated with lower risk of EC [8, 9]. It is reasonable to speculate that omega-3 PUFAs might have the potential to improve survival outcomes of EC patients.

However, epidemiological evidences of dietary intake of omega-3 PUFAs were inconsistent [10, 11]. A meta-analysis also found a positive association between dietary omega-3 PUFAs and EC risk in Asian [12]. Findings from previous case-control studies of Chinese also suggested that PUFA intake was associated with increased EC risk [13, 14]. Fatty acids levels in these studies were calculated from dietary questionnaires, which were subject to recall bias and measurement error. Instead, plasma PUFAs level act as a more objective and precise biomarker of PUFAs intake [15], which also reflect endogenous PUFAs metabolism, including synthesis and desaturation/elongation [16]. To our knowledge, only one study reported the association between fatty acids and EC risk based on biospecimen [17]. However, the study only reported 8 fatty acids, including 3 saturated fatty acids, 2 monounsaturated fatty acids and 3 omega-6 PUFAs. The association of EC risk and omega-3 PUFAs was unknown. In addition, no study reported the relationship between omega-3 PUFAs and EC survival.

Therefore, we examined plasma omega-3 PUFAs (ALA, EPA, DPA, DHA, total omega-3 PUFA) in EC patients, aiming to explore the associations of plasma omega-3 PUFAs with survival outcomes (recurrence or mortality) in Chinese women.

## Methods

### Patients

All women with histologically confirmed EC who underwent surgery in Fudan University Shanghai Cancer Center between July 2008 and November 2013 were eligible for this study. In total, 202 patients with enough plasma samples at time of surgery were included. Blinded to exposure data, physicians retrospectively reviewed the clinical and medicine records of all the patients. Pathological tumor stage was identified accroding to International Federation of Gynecology and Obstetrics (FIGO) 2009 staging [18]. Fasting blood samples were collected before surgery in hospital. Plasma was separated and stored at − 80 °C until fatty acids were measured. The survival condition after surgery was acquired through medical record and phone interview by September 2, 2017. Seven patients were lost to follow-up and were excluded from the study. In total, 195 participants were included in the final analysis (Supplemental Figure 1). The study protocol was approved by the Ethical Review Committee of the Fudan University Shanghai Cancer Center. All participants provided informed consents.

### Covariate assessments

Demographic characteristics (including age, race, education, occupation, et, al), smoking status, drinking status, history of disease, long-term medication history (took medicine medicines treating diabetes, hypertension, and hypothyroidism for more than 3 months), family history of cancer, reproductive history (including times of pregnancy, abortion, premature delivery, term delivery), menstrual history (including menophania, menstrual period, menopausal status, age at menopause), exogenous hormone use, and medical conditions were collected prior to surgery by physicians. Anthropometric characteristics (including weight and height) were measured during the examination upon admission to hospital. None of the patients had current smoking, and only one patient had self-reported current drinking.

### Ascertainment of end points

The endpoint of this study was the relationship between n-3 PUFA and recurrence of EC. We assessed recurrence of EC, which ever came first for additional analysis. The recurrences and deaths were ascertained from family members and medical records. Time to event (in months) was defined as the difference between date of surgery and date of recurrence, date of death, date last seen, or September 2nd 2017. Patients who didn’t have endpoint events were defined as censored.

### Plasma fatty acids measurement

The method for plasma fatty acids measurement has been described as previously [19]. Briefly, 0.8 mg nonadecanoic acid methyl ester (C19:0), chosen as the internal standard for quantification, was added to the 100 μl plasma sample. Fatty acids were extracted by dichloromethane/methanol from the mix sample, and then were incubated with mixture of sulfuric acid and methanol for fatty acid methyl esters (FAMEs). FAMEs were extracted again using n-hexane after methylation and then re-dissolved in isooctane. FAMEs were finally separated by gas chromatography (Agilent 6890 GC; SP-2560 capillary column: 100 m × 0.25 mm inside diameter × 0.20um thickness) and detected by flame ionization detector (FID). Individual FAME was identified according to remain time of its standard substance. Data of each fatty acid was expressed as percentage of total fatty acids. Two quality control (QC) samples, which come from a homogeneous pool of plasma, were inserted in every 20 samples as a batch. The coefficient of variation (CVs) of QC were 5.4% for ALA, 10.6% for EPA, 11.3% for DPA, and 12.2% for DHA. Highly unsaturated Fatty Acid (HUFA) was defined as sum of EPA, DPA, DHA. Total omega-3 PUFA was defined as sum of ALA, EPA, DPA, and DHA. The ratio of EPA/ALA was used as a proxy for coupling Δ6- desaturase and fatty acid elongase 5 activity [20]. The ratio of DPA/EPA was used to reflect the activity of fatty acid elongase 5/2 [20].

### Statistical analysis

Wilcoxon’s rank sum test for continuous variables and Chi-Square test for categorical variables were applied to compare the basic characteristics between non-recurrence and recurrence populations, and those between total analysis and non-accessible populations. Levels of ALA, EPA, DPA, DHA, HUFA, total omega-3 PUFA, EPA/ALA, and DPA/EPA were classfied into tertiles as indicator variables. *P* values for continuous variables such as age at diagnosis, body mass index (BMI), age of menarche, and age at menopause were obtained from linear regression with tertiles. *P* values for categorical variables were obtained from Mantel-Haenszel chi-square test.

Kaplan-Meier curve was constructed to describe survival status. Cox proportional hazards model was used to calculate the hazard ratios (HRs) and 95% confidence intervals (CIs). We selected the covariates adjusted in cox models based on a priori risk factors for EC, including age, BMI, stage, hormone therapy, and adjuvant therapy. The proportional hazards assumption was verified by testing the association of Schoenfeld residuals and survival time. All the models complied with the assumption of cox model. Covariates variables with missing data were coded as indicator variables, continuous variables with missing data were assigned with the median value (*n* = 2 for times of pregnancy only). The omega-3 PUFAs were divided into binary variable according to median to fit Cox models. Potential nonlinear associations were evaluated using restricted cubic spline (RCS) regression by treating omega-3 PUFAs as continuous variables in the cox proportional hazard models, with 5 knots at 5th, 25th, 50th, 75th, and 95th percentiles.

Stratified analysis were conducted by a priori factors, including age at diagnosis (< 55, ≥55), BMI (< 24, ≥24), age at menarche (< 15, ≥15), menopausal status (premenopausal, postmenopausal), age at menopause (< 50, ≥50), gravidity (< 3, ≥3), long-term medication history (yes, no), stage (I-II, III-IV), grade (1–2, 3), myometrial invasion (< 1/2, ≥1/2), cervical gland involvement (yes, no), and cervical stroma involvement (yes, no). Limited to the sample sizes of events, cox regression with Firth’s Penalized Likelihood was used to estimate the HR and 95%CI for each strata. Models were adjusted for age, BMI, and stage only. *P*-trend values were obtained by treating ordinal omega-3 PUFAs as continuous variable in cox regression models. Interaction was tested by entering the cross product of two variables of interest. Likelihood ratio tests were used to estimate *P* value of interactions. All analyses were performed using SAS 9.3 software and R software. All *P*-values were two-sided, and *P* < 0.05 was considered statistically significant.

## Results

### Basic characteristics of participants at time of surgery

Among the 195 eligible patients with EC, we documented 13 recurrences and 11 deaths, of which, 2 deaths were attributed to EC. The median (interquartile range (IQR)) age of patients at time of diagnosis was 55 (50–61) years old. Compared to non-recurrence patients, those with recurrent disease had higher proportion of ever hormonal therapy, higher stage, lower grade, deeper myometrial invasion, positive extra-uterine involvement, positive lymph node involvement, and ever adjuvant treatment (Supplemental Table 1). Patients with higher plasma total omega-3 PUFA tended to experience menopause earlier (*P* = 0.05) (Table 1).

**Table 1:** Basic characteristics of patients with endometrial cancer according to tertiles of plasma total omega-3 PUFA^a^

Variable	Tertile1	Tertile2	Tertile3	P^g^
Total omega-3 PUFA (wt%)	< 4.03	4.04–4.99	> 5.00	
No. of patients	65	66	64	
Age at diagnosis	56.0(49.0–64.0)	57.0(50.0–61.0)	54.0(51.0–58.0)	0.28
Body mass index (kg/m2)	24.2(21.6–27.0)	24.0(21.8–27.3)	23.6(21.3–26.7)	0.36
Age at menarche, years	15.0(14.0–16.0)	15.0(14.0–17.0)	15.0(14.0–16.0)	0.49
Menopausal status				0.80
Premenopausal	22(33.85)	20(30.3)	23(35.94)	
Postmenopausal	43(66.15)	46(69.70)	41(64.06)	
Age at menopause, years	51.0(49.0–53.0)	50.0(49.0–53.0)	50.0(48.0–52.0)	0.056
Gravidity				0.94
0–2	28(43.08)	28(43.94)	28(43.75)	
≥3	37(56.92)	37(56.06)	36(56.25)	
Full-term birth				0.59
0–2	45(69.23)	50(75.76)	47(73.44)	
≥3	20(30.77)	16(24.24)	17(26.56)	
Abortion (yes), %	37(56.92)	39(59.09)	39(60.94)	0.64
Hormonal therapy (ever), %	3(4.62)	3(4.55)	6(9.38)	0.48
Hypertension (ever), %	15(23.08)	16(24.24)	11(17.19)	0.42
History of other cancer (ever), %^b^	2(3.08)	3(4.55)	6(9.38)	0.12
Family history of cancer (ever), %^c^	17(26.15)	17(25.76)	11(17.19)	0.23
Long-term medication history (ever), %^d^	9(13.85)	15(22.73)	11(17.19)	0.66
FIGO stage				0.85
I-II	53(81.54)	55(83.33)	53(82.81)	
III-IV	12(18.46)	11(16.67)	11(17.19)	
Grade				0.86
Low-grade (1–2)	54(83.08)	58(89.23)	53(84.13)	
High-grade (3)	11(16.92)	7(10.77)	10(15.87)	
Myometrial invasion				0.92
< 50%	49(75.38)	46(71.88)	48(76.19)	
≥50%	16(24.62)	18(28.13)	15(23.81)	
Histological type				0.69
Endometrioid adenocarcinoma	62(95.38)	61(93.85)	60(93.75)	
Others	3(4.62)	4(6.15)	4(6.25)	
Extrauterine Involvement^e^				0.95
Negative	59(90.77)	58(90.63)	57(90.48)	
Positive	6(9.23)	6(9.38)	6(9.52)	
Lymph node involvement^f^				0.78
Negative	56(86.15)	55(83.33)	56(87.5)	
Positive	6(9.23)	5(7.58)	4(6.25)	
Unknown	3(4.62)	6(9.09)	4(6.25)	
Adjuvant treatment				0.86
None	41(63.08)	44(66.67)	39(60.94)	
Chemotherapy or radiotherapy	20(30.77)	20(30.30)	22(34.38)	
Unknown	4(6.15)	2(3.03)	3(4.69)	

^a^Total plasma omega-3 PUFA was defined as the sum of ALA, EPA, DPA and DHA. Data are presented as proportions for categorical data, medians and interquartile ranges for continuous data

^b^History of other cancer, including breast cancer, thyroid cancer, rectal cancer

Long-term medication history, including anti-cancer drugs, blood pressure drugs, diabetes

^c^Family history of cancer: immediate relatives who have a history of any cancer were marked as yes, the cancer including Lung cancer, gastric cancer, colorectal cancer, endometrial cancer, liver, gallbladder, ovarian cancer, esophageal cancer

^d^Long-term medication history, including anti-cancer drugs, blood pressure drugs, diabetes

^e^ExtrauterineInvolvement: any of the adnexa or vagina or parametrialwas involved

^f^Lymph node involvement: any of the Pelvic lymph node involvement or Para-aortic lymph node involvement was involved

^g^P values were obtained using the Chi-Square test for categorical data and linear regression for continuous data

### Omega-3 PUFAs and recurrence

**Fig. 1:**
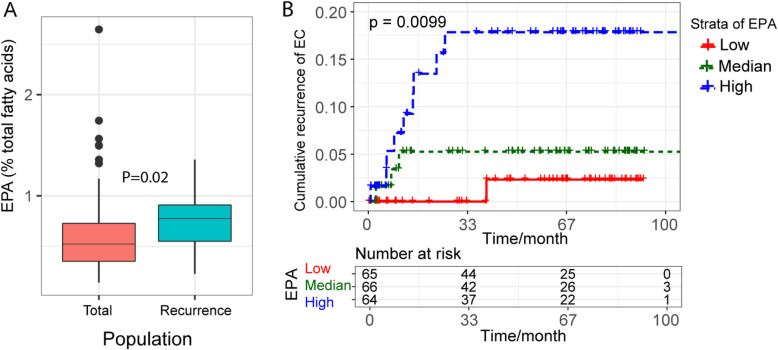
The association between level of EPA and EC recurrence. **a** Level of plasma EPA between non-recurrence population and recurrence population. Kruskal-Wallis was used to test the difference EPA level between these two groups, *P* < 0.05 was considered as significant. **b** Kaplan-Meier curves of EC recurrence according to tertile of plasma EPA. Blue line: low level of EPA (first tertile); Green line: median level of EPA (second tertile); Red line: high level of EPA (third tertile), *P* < 0.05 was considered as significant

The median time between EC surgery and last time of follow up was 58 months (IQR:11.7–222.6 months). Compared to non-recurrence patients, patients with EC recurrence had higher level of EPA (Fig. 1, Supplemental Table 2). The Kaplan-Meier curves also showed that patients who had higher level of plasma EPA tended to have a higher risk of EC recurrence (*P* for log-rank test = 0.009) (Fig. 1). HR estimates of EC recurrence according to tertiles of plasma omega-3 PUFAs were showed in Table 2. Compared to the lowest tertile (T1), patients in highest tertile (T3) of plasma EPA had higher risk of EC recurrence (HR_T3vsT1_ = 9.71; CI = 1.23–76.73; *P*-trend = 0.01). After further adjusted for BMI, stage, hormone therapy, and adjuvant therapy, *P* trend was still significant (HR_T3vsT1_ = 6.02; CI = 0.7–52.06; *P*-trend = 0.04). None of the other omega-3 PUFAs was significantly associated with EC recurrence. The significant association was also observed when EPA was classified dichotomies according to median (Supplemental Table 3). No significant non-linear associations was observed between EC recurrence and any omega-3 PUFAs either (*P* > 0.05, data not shown).

**Table 2:** Associations between plasma n-3 PUFAs and endometrial cancer recurrence^a^

Variable	Tertile1	Tertile2	Tertile3	P-trend^b^
C18:3n3				
wt%	0.62(0.54–0.67)	0.86(0.79–0.93)	1.23(1.07–1.33)	
Events/total(n/n)	3/65	5/66	5/64	
Model 1	1.00	1.87(0.43–8.1)	1.94(0.45–8.37)	0.43
Model 2	1.00	1.74(0.41–7.4)	3.78(0.87–16.41)	0.07
Model 3	1.00	2.15(0.48–9.66)	3.28(0.7–15.41)	0.13
C20:5n3				
wt%	0.31(0.26–0.36)	0.53(0.46–0.6)	0.84(0.73–1.02)	
Events/total(n/n)	1/65	3/66	9/64	
Model 1	1.00	2.88(0.3–27.74)	9.71(1.23–76.73)	0.01
Model 2	1.00	2.83(0.29–27.34)	7.3(0.89–59.68)	0.03
Model 3	1.00	1.98(0.19–21.12)	6.02(0.7–52.06)	0.04
C22:5n3				
wt%	0.39(0.35–0.42)	0.5(0.46–0.54)	0.63(0.6–0.7)	
Events/total(n/n)	1/65	6/66	6/64	
Model 1	1.00	6.57(0.78–55.51)	6.61(0.79–55.1)	0.1
Model 2	1.00	8.48(0.99–72.72)	7.82(0.93–65.77)	0.06
Model 3	1.00	7.02(0.73–67.61)	7.19(0.81–64.1)	0.1
C22:6n3				
wt%	2.04(1.74–2.21)	2.62(2.47–2.82)	3.39(3.11–4)	
Events/total(n/n)	2/65	7/66	4/64	
Model 1	1.00	3.14(0.65–15.13)	1.97(0.36–10.86)	0.6
Model 2	1.00	3.25(0.66–15.89)	1.59(0.29–8.82)	0.87
Model 3	1.00	3.25(0.61–17.31)	1.22(0.2–7.54)	0.77
HUFA				
wt%	2.88(2.61–3.06)	3.65(3.48–3.84)	4.8(4.31–5.67)	
Events/total(n/n)	2/65	4/66	7/64	
Model 1	1.00	1.77(0.32–9.67)	3.56(0.73–17.29)	0.09
Model 2	1.00	1.69(0.3–9.42)	2.55(0.52–12.59)	0.23
Model 3	1.00	1.57(0.27–9.14)	2(0.38–10.54)	0.42
Total n-3 PUFA				
wt%	3.68(3.42–3.93)	4.53(4.35–4.72)	5.79(5.22–6.65)	
Events/total(n/n)	3/65	3/66	7/64	
Model 1	1.00	0.95(0.19–4.72)	2.4(0.62–9.37)	0.15
Model 2	1.00	0.96(0.19–4.83)	2.3(0.59–9.02)	0.17
Model 3	1.00	0.77(0.14–4.19)	1.7(0.4–7.33)	0.32

*Abbreviation*: *PUFA* Polyunsaturated Fatty Acids, *HUFA* Highly Unsaturated Fatty Acid

^a^HUFA was defined as EPA + DPA + DHA. Total omega-3 PUFA was defined as ALA+EPA + DPA + DHA. Cox regression was used to estimate the HRs and CIs. PUFAs were classified into three groups based on the tertiles in total subjects, the lowest tertile group was treated as reference

^b^P-trend values were conducted by assigning the median value to each tertile in the Cox regression models

Model 1: adjusting for age

Model 2: adjusting for age, BMI, and stage

Model 3: adjusting for age, BMI, stage, hormone therapy, and adjuvant therapy

### Omega-3 PUFAs and recurrence within subgroups

**Fig. 2:**
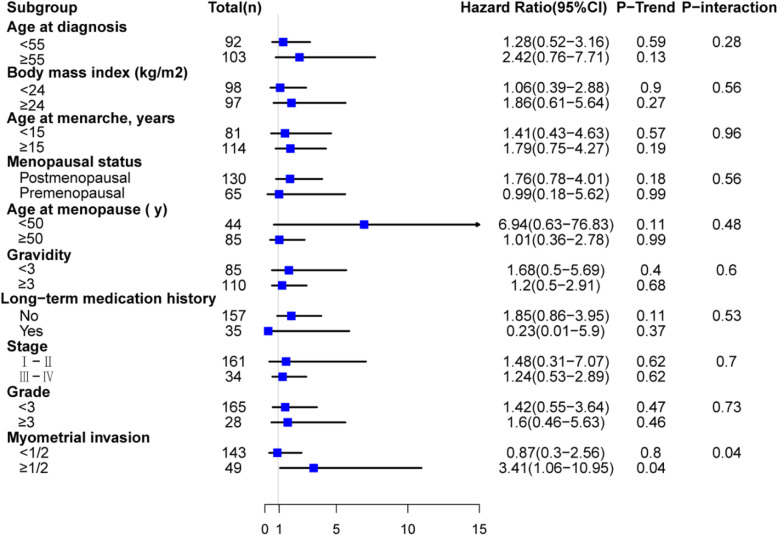
Stratified analysis of the association of total omega-3 PUFAs and the risk of EC recurrence. All the HRs were adjusted for age, bmi, and stage in cox regression with Firth’s Penalized Likelihood, P-trend values were obtained by treating ordinal omega-3 PUFA as continuous in cox regression models in subgroups. Interaction test was conducted by entering the cross product of total omega-3 PUFA and the variables of interest. *P* < 0.05 was considered as significant

We explored the modification effect of potential a priori risk factors on the relationship between plasma omega-3 PUFAs and cancer recurrence with stratified analysis. In the patients with higher-stage, lower-grade, and positive ER, higher plasma EPA was associated with higher risk of EC recurrence (Supplemental Table 4). The positive association between total omega-3 PUFA and risk of EC recurrence trended to be stronger in patients who had deeper myometrial invasion (OR = 3.41; 95% CI = 1.06–10.95, *P*-interaction = 0.04) (Fig. 2). Positive associations with risk of EC recurrence were found for ALA in postmenopausal patients (OR = 2.32; 95% CI = 1.05–5.14); for EPA in patients without long-term medication (OR = 2.87; 95% CI = 1.14–7.23); for DPA in patients with older age at diagnosis (OR = 5.84; 95% CI = 1.24–27.54) and higher-stage (OR = 3.02; 95% CI = 1.11–8.25). No significant interaction was observed for these omega-3 PUFAs (Supplemental Figure 2).

### The ratios of omega-3 PUFAs and recurrence

The ratio of EPA/ALA could reflect the activity of fatty acid desaturase 6 and fatty acid elongase 5, and the ratio of DPA/EPA could reflect the activity of fatty acid desaturase 5/2. A negative association was observed for EC recurrence with DPA/EPA ratio (*P*-trend = 0.027) (Table 3).

**Table 3:** Association between ratios of omega-3 PUFAs and EC recurrence^a^

Variable	Tertile1	Tertile2	Tertile3	P-trend^b^
C20:5n3/C18:3n3	0.37(0.3–0.42)	0.59(0.53–0.65)	1.07(0.86–1.39)	
Events/total(n/n)	4/65	4/66	5/64	
Model 1	1.00	1.04(0.26–4.16)	1.21(0.32–4.58)	0.805
Model 2	1.00	0.82(0.2–3.36)	0.65(0.16–2.67)	0.541
Model 3	1.00	0.49(0.1–2.44)	0.5(0.11–2.24)	0.505
C22:5n3/C20:5n3	0.66(0.56–0.73)	1(0.92–1.07)	1.44(1.3–1.73)	
Events/total(n/n)	6/65	3/66	2/64	
Model 1	1.00	0.46(0.14–1.53)	0.13(0.02–1.03)	0.027
Model 2	1.00	0.96(0.26–3.6)	0.16(0.02–1.3)	0.074
Model 3	1.00	0.90(0.23–3.44)	0.14(0.02–1.17)	0.056

^a^Cox regression was used to estimate the HRs and CIs. PUFAs were classified into three groups based on the tertiles in total subjects, the lowest tertile group was treated as reference

^b^P-trend values were conducted by assigning the median value to each tertile in the Cox regression models

Model 1: adjusting for age

Model 2: adjusting for age, BMI, and stage

Model 3: adjusting for age, BMI, stage, hormone therapy, and adjuvant therapy

## Discussion

In the current study, we observed that higher plasma EPA was associated with higher risk of EC recurrence. Other omega-3 PUFAs, including ALA, DPA, DHA, were not significantly associated with recurrence of EC. Age at diagnosis, menopause status, gravidity, stage, and grade did not show significant modification effects on the associations between omega-3 PUFAs and recurrence.

Up to 9 years follow up, the recurrence rate in our study was 6.7%. some other cohort studies reported that recurrence rate of EC ranged from 8 to 19% [21, 22]. However, the rate of a Danish cohort (7%) was near to us [23]. The population of this study consisted of early-stage EC patients, and most patients (83%) in our current study were in I-II stage. EC patients detected at an earlier stage tend to have better prognosis. 74–91% of patients at early stage of EC had a 5-year survival; whereas the percentage of patients in late stage EC with a 5-year survival was only 20–63% [24]. It is worth noting that some patients accepted modified radical hysterectomy or radical hysterectomy in the current study. Different recurrence rates may also be due to differences in ethnics, lifestyle, therapeutic methods and lengths of follow up.

We found that higher plasma EPA was associated with higher recurrence risk of EC. This is consistent with a report by Brasky et al. in the VITAL cohort [10], where the highest quintile of dietary EPA + DHA was associated with 79% increased EC risk. A report from the Iowa women’s health study also showed that fish intake elevated 40% risk of EC [25]. Xu et al. reported that fish intake was positively associated with EC (OR = 2.4, 95%CI = 1.8–3.1, quintile 4 compared to quintile 1) [14], which was in line with our results. However, results of other studies were different. Two other studies reported significant decreased EC risk with fish intake [26, 27]. The inconsistency may be attributed to different cooking methods. Stir frying, the most popular cooking style in China may attribute production of mutagens and carcinogens [8, 13]. In addition, higher intake of salted fish in Chinese, which may be accompany with N-nitrosamines [8, 12, 13], may also contribute to increase EC risk. Therefore, higher intake of omega-3 PUFAs, as a surrogate of fish intake, may go along with a higher cancer risk in Chinese.

We found that plasma EPA was higher in subjects with high-stage and low differentiated adenocarcinomas of EC. Some studies also reported that EPA may facilitate tumors’ progression to aggressive or high-grade type [28, 29]. We speculate the possible reasons are as following:1) EPA may promote free radicals production and oxygens activation in the beta-oxidative reaction, which subsequently increases DNA mutation and cause carcinogenesis [30]. 2) Higher dose of omega-3 PUFAs may suppress CD8+ T cells, subsequently maintaining the tumor growth in cancer immunosurveillance via elevated MDSCs levels [31]. 3) Higher omega-3 PUFAs intake may increase sex hormone, including estradiol and estrone, in circulation, which results in endometrial proliferation. A randomized trial showed that fish oil supplementation increased serum estradiol and estrone in premenopausal women [32] and urinary estrone in postmenopausal women [33]. Therefore, plasma omega-3 PUFA may be higher in patients with higher grade of cancer. 4) PUFAs can serve as substrates for energy production or components of membranes for tumor cell growth, A positive association between ALA and EC recurrence (T3 vs T1 OR = 3.28) was observed, which not statistically significant possibly due to small sample size. This might be in line with the positive association between EPA and EC recurrence as EPA could also be synthesized from ALA. The underlining mechanisms could be that ALA interferes with 5a-reductase and affects fatty acids oxidation to form free radicals [34]. ALA may also link to aggressive cancer through activating cell signaling pathways, like MEK1 and MEKK1 pathways [35].

**Fig. 3:**
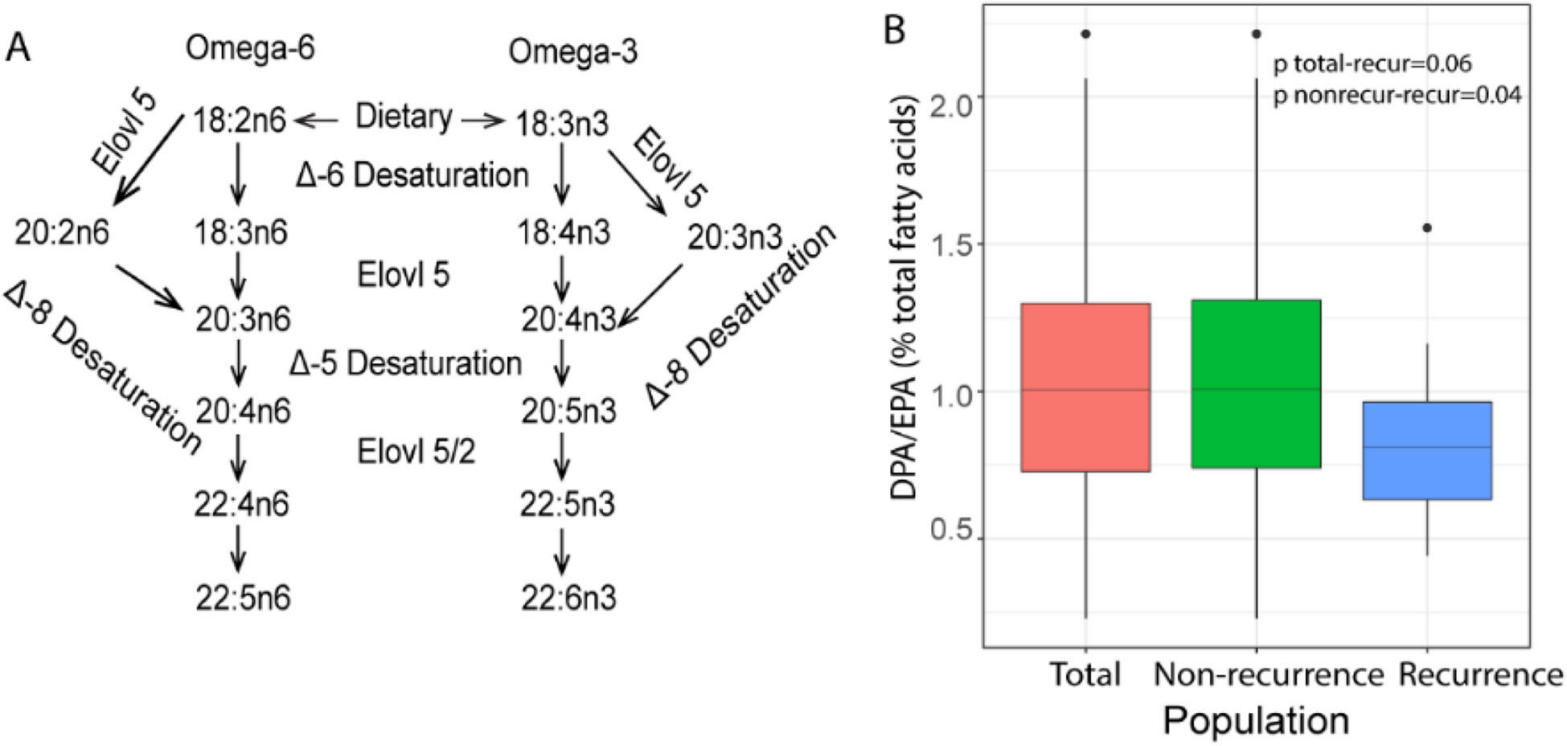
Omega-3 PUFA metabolism is related with EC recurrence. **a** Pathway of omega-3 PUFAs metabolism. **b** Elongase 5/2 activity was calculated as the ratio of DPA/EPA. Level of DPA/EPA among total population, non-recurrence and recurrence population. Kruskal-Wallis was used to test the difference DPA/EPA level among these three groups, *P* < 0.05 was considered as significant

Plasma omega-3 PUFAs levels are determined by dietary intake and endogenous synthesis through elongation and desaturation from ALA (C18:3n3) [36]. Lipogenesis is activated in cancer cell [37], and omega-3 PUFA metabolism (Fig. 3) may be disordered in cancer patients. The intermediates C18:4 and C20:4 are always present at very low levels and were under detective level in our present measurements. Though no significant association was observed between the EPA/ALA and EC recurrence, a marginally lower level of DPA/EPA was associated with increased EC recurence, suggesting decreased activity of elongase 5/2 in recurrence patients. This result was in line with a study which reported that elongase 5/2 elongase activity was higher in non-progressed cancer group than progressed cancer group [38]. Higher level of EPA/DPA in recurrence patients than total patients in our study might suggest that accumulation of EPA was much greater than conversion from EPA to DPA. However, it is difficult to disentangle exogenous intake and endogenous synthesis of omega-3 PUFA. More experimental studies are needed to explore the role of elongase 5/2 in EC recurrence.

We also estimated the modification effects of several a priori factors on the associations between omega-3 PUFAs and EC recurrence. We found that the association between total omega-3 PUFA and EC recurrence was stronger among patients with deeper myometrial. Besides, the association between individual omega-3 PUFA and EC was stronger in patients with higher-stage, higher-grade, postmenopause, or older age. These modified factors were well accepted prognostic factors for endometrial carcinoma [39]. The results were also consistent with previous study which reported that advanced stage and older age were associated with worse prognosis [39]. BMI was reported as a modifying factor for EC. However, we didn’t observe significant interaction of omega-3 PUFA and BMI on the EC recurrence, which might be due to small number of recurrent events and limited statistical power.

To our knowledge, this is the first report examining the associations of plasma omega-3 PUFAs and EC prognosis. The major strength of our study was that plasma PUFA levels were used as objective biomarkers of fatty acid status and irrespective of the source and inherent variability of dietary questionnaires. About 83% of patients in our study were diagnosed at early stage EC, and surgery alone was the standard of treatment, which maintained our subjects with higher homogeneity. Several limitations should be considered too. First, plasma omega-3 PUFAs were only measured once at baseline, which may change during the follow-up period as EC progressed. Second, the relatively few events of EC recurrences and deaths in our study led to the restricted statistical power to conduct stratified analysis. In addition, unknown confounders were not fully controlled in the models. Additionally, the BMI range of our population was much lower compared with those from Western countries, which might limit the applicability of our results to other countries. Further studies on populations with wider range of BMI will be needed.

## Conclusions

We observed a positive association between plasma EPA and recurrence of EC in Chinese women. Further studies are warranted to confirm these findings and explore the underlying mechanisms.

## Supplementary information

**Additional file 1.**

## Data Availability

The datasets used and/or analysed during the current study are available from the corresponding author on reasonable request.

## Abbreviations

EC: Endometrial cancer
ALA: Alpha-linolenic acid
EPA: Eicosapentaenoic acid
DPA: Docosapentaenoic acid
DHA: Docosahexaenoic acid
PUFA: Polyunsaturated fatty acid
