# Supplementary material for: Plasma omega-3 polyunsaturated fatty acids and recurrence of endometrial cancer
Source: BMC Cancer. 2020 Jun 20;20:576. doi: 10.1186/s12885-020-07035-5 (PMC7305622; doi:10.1186/s12885-020-07035-5)
Supplement: Supplementary file 1 — Additional file 1. [file 12885_2020_7035_MOESM1_ESM.docx]

**Supplemental Figure 1** Flowchart of study participants’ selection in this study.


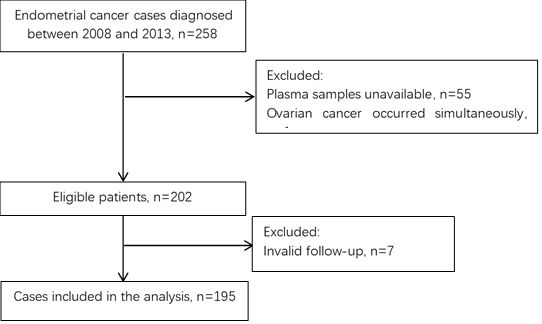


**Supplemental Figure 2** Kaplan-Meier curves of EC recurrence according to tertile of plasma ALA, DPA, DHA and total omega-3 PUFA.

Low: the first tertile median: the second tertile high: the third tertile

**
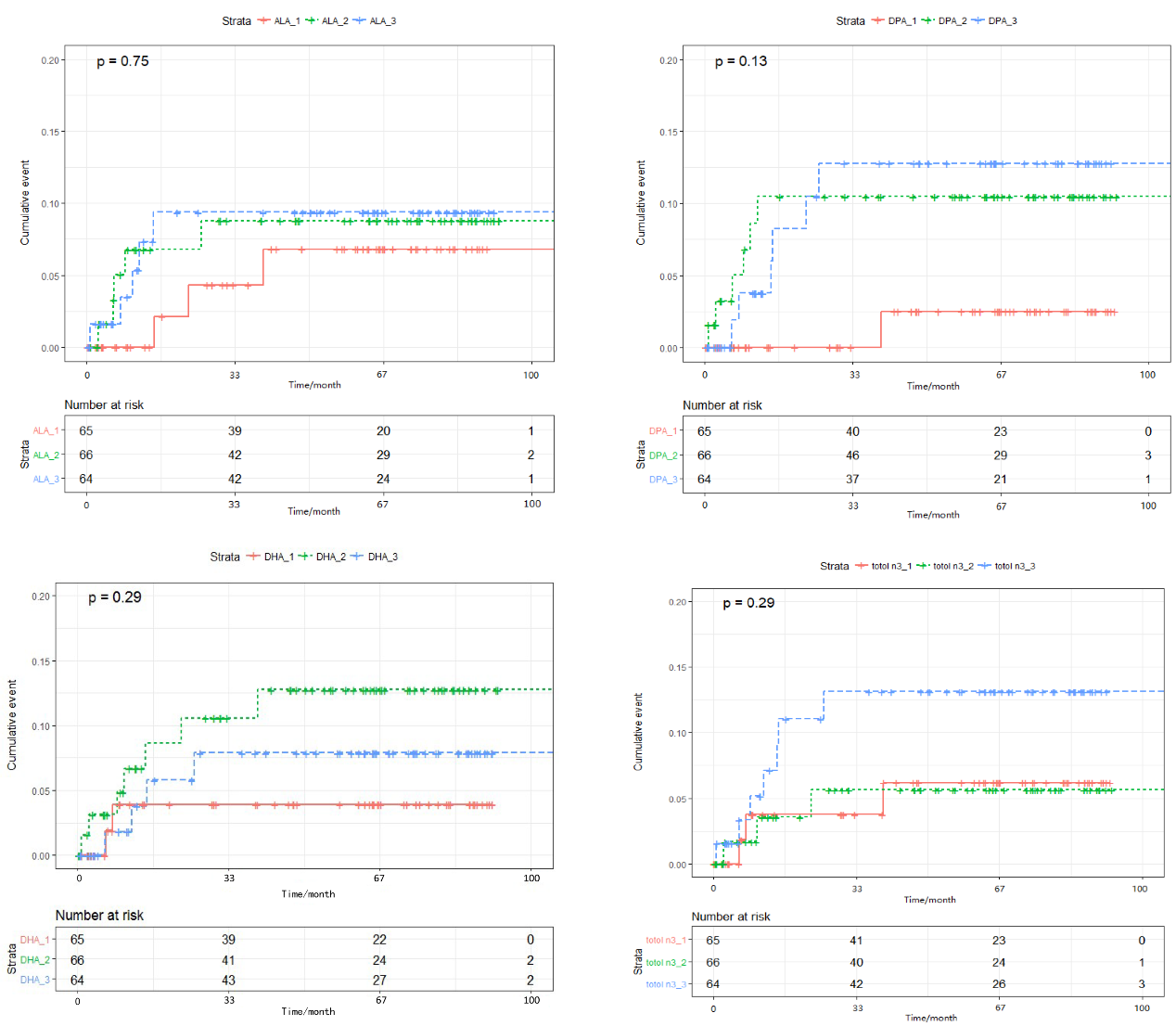
**

**Supplemental Table1**. Basic characteristics of patients with endometrial cancer between the no-recurrence and recurrence population.

|  |  | no-recurrence (n=182) | recurrence(n=13) | P |
| --- | --- | --- | --- | --- |
| Age at diagnosis |  | 55.0(50.0-60.0) | 54.0(52.0-63.0) | 0.28 |
| Body mass index (kg/m2) |  | 23.9(21.7-27.1) | 21.8(18.9-24.2) | 0.07 |
| Education, n (%) |  |  |  | 0.36 |
| ≤Primary School graduate |  | 15（8.24） | 1(7.69) |  |
| Middle School |  | 16（8.79） | 0 |  |
| High School |  | 2（1.10） | 1(7.69) |  |
| College or advanced degree |  | 7（3.85） | 0 |  |
| Unknown |  | 142（78.02） | 11(84.6) |  |
| Age at menarche, years |  | 15.0(14.0-16.0) | 16.0(14.0-17.0) | 0.14 |
| Menopausal status |  |  |  | 0.23 |
| Premenopausal |  | 63(34.62) | 2(15.38) |  |
| Postmenopausal |  | 119(65.38) | 11(84.62) |  |
| Age at menopause ( y) |  | 50.0(48.0-53.0) | 50.0(48.0-51.0) | 0.20 |
| Gravidity |  |  |  | 0.78 |
| 0-2 |  | 80(43.96) | 5(38.46) |  |
| ≥3 |  | 102(56.04) | 8(61.54) |  |
| premature birth |  |  |  | 0.13 |
| 0 |  | 181(99.45) | 12(92.31) |  |
| ≥1 |  | 1(0.55) | 1(7.69) |  |
| Abortion |  |  |  | 1.00 |
| no |  | 75(41.21) | 5(38.46) |  |
| yes |  | 107(58.79) | 8(61.54) |  |
| Full-term birth |  |  |  | 0.75 |
| 0-2 |  | 133(73.08) | 9(69.23) |  |
| ≥3 |  | 49(26.92) | 4(30.77) |  |
| hormonal therapy |  |  |  | 0.004 |
| Never |  | 163（89.56） | 7（53.85） |  |
| ever |  | 7（3.85） | 5(38.46) |  |
| Unknown |  | 12(6.59) | 1(7.69) |  |
| Hypertension |  |  |  | 1.00 |
| Never |  | 143(78.57) | 10(76.92) |  |
| ever |  | 39(21.43) | 3(23.08) |  |
| History of other cancer |  |  |  | 0.54 |
| No |  | 172(94.51) | 12(92.31) |  |
| Yes |  | 10(5.49) | 1(7.69) |  |
| Family history of cancer |  |  |  | 0.50 |
| No |  | 141(77.47) | 9(69.23) |  |
| Yes |  | 41(22.53) | 4(30.77) |  |
| Long-term medication history |  |  |  | 1.00 |
| Never |  | 146(80.22) | 11(84.62) |  |
| ever |  | 33(18.13) | 2(15.38) |  |
| Unknown |  | 3(1.65) | 0 |  |
| FIGO stage |  |  |  | 1.483E-06 |
| Ⅰ-Ⅱ |  | 158(86.81) | 3(23.08) |  |
| Ⅲ-Ⅳ |  | 24(13.19) | 10(76.92) |  |
| Grade |  |  |  | 0.03 |
| low-grade(1-2) |  | 157(87.22) | 8(61.54) |  |
| high-grade(3) |  | 23(12.78) | 5(38.46) |  |
| Myometrial invasion |  |  |  | 0.005 |
| <50% |  | 138(77.09) | 5(38.46) |  |
| ≥50% |  | 41(22.91) | 8(61.54) |  |
| Extrauterine Involvement |  |  |  | 0.03 |
| Negative |  | 165(92.18) | 9(69.23) |  |
| Positive |  | 14(7.82) | 4(30.77) |  |
| lymph node involvement |  |  |  | 0.004 |
| Negative |  | 160(87.91) | 7(53.85) |  |
| Positive |  | 11(6.04) | 4(30.77) |  |
| unknown |  | 11(6.04) | 2(15.38) |  |
| Adjuvant treatment |  |  |  | 2.592E-04 |
| None |  | 122(67.03) | 2(15.4) |  |
| Chemotherapy or radiotherapy |  | 51(28.02) | 11(84.6) |  |
| unknown |  | 3(4.95) | 0 |  |
| ER |  |  |  | 0.34 |
| Negative |  | 17（9.34） | 2(15.38) |  |
| Positive |  | 123（67.58） | 10(76.92) |  |
| unknown |  | 42(23.08) | 1(7.69) |  |
| PR |  |  |  | 0.27 |
| Negative |  | 14(7.69) | 2(15.38) |  |
| Positive |  | 126(69.23) | 10(76.92) |  |
| unknown |  | 42(23.08) | 1(7.69) |  |
| Maximum diameter of tumor |  | 2.7（2.0-3.5） | 3.5(3.0-4.5) | 0.01 |

P value were obtained from Wilcoxon rank test for continuous variables and chi-square test for categorical variables

None of the patients had current smoking; only one woman had current drinking. All the recurrence women had child bearing history and without Diabetes history.

Extrauterine Involvement: any of the Adnexa or Vagina or Parametrial was Involved

Lymph node involvement: any of the Pelvic lymph node involvement or Para-aortic lymph node involvement was involved

History of other cancer, including breast cancer, thyroid cancer, rectal cancer

Family history of cancer: immediate relatives who have a history of cancer were marked as yes, the cancer including Lung cancer, gastric cancer, colorectal cancer, endometrial cancer, liver, gallbladder, ovarian cancer, esophageal cancer

Long-term medication history, including anti-cancer drugs, blood pressure drugs, diabetes

**Supplemental Table 2** Plasma PUFA levels among the total, non-recurrence, and recurrence population.

|  | Total | Non-recurrence | recurrence | P^a^ | P^b^ |
| --- | --- | --- | --- | --- | --- |
|  | n=195 | n=182 | n=13 |  |  |
| C18:3n3 | 0.86(0.67-1.07) | 0.83(0.67-1.06) | 0.93(0.88-1.19) | 0.439 | 0.408 |
| C20:5n3 | 0.52(0.36-0.72) | 0.51(0.36-0.69) | 0.78(0.56-0.91) | 0.022 | 0.015 |
| C22:5n3 | 0.49(0.42-0.6) | 0.49(0.42-0.59) | 0.54(0.47-0.7) | 0.112 | 0.09 |
| C22:6n3 | 2.62(2.21-3.1) | 2.6(2.21-3.1) | 2.84(2.59-3.37) | 0.357 | 0.325 |
| HUFA | 3.62(3.06-4.29) | 3.59(3.03-4.18) | 4.07(3.36-4.71) | 0.161 | 0.134 |
| Total n-3PUFA | 4.53(3.93-5.21) | 4.5(3.91-5.17) | 5(4.31-5.9) | 0.106 | 0.084 |
| n-6 | 40.72(37.78-43.08) | 40.47(37.88-43.1) | 41.12(35.88-42.65) | 0.777 | 0.762 |
| AA | 6.86(5.87-8.13) | 6.85(5.8-8.13) | 7.25(6.13-7.94) | 0.737 | 0.72 |
| AA/HUFA | 1.89(1.53-2.36) | 1.9(1.53-2.36) | 1.8(1.45-2.06) | 0.468 | 0.438 |
| n-6/n-3 | 8.87(7.63-10.42) | 8.91(7.71-10.64) | 8.2(7.02-9.89) | 0.2 | 0.17 |
| C20:5n3/ C18:3n3 | 0.58(0.42-0.86) | 0.57(0.42-0.84) | 0.71(0.46-1.44) | 0.179 | 0.151 |
| C22:5n3/ C20:5n3 | 1.004(0.73-1.3) | 1.01(0.74-1.31) | 0.81(0.63-0.96) | 0.059 | 0.044 |

P^a^ obtained from Wilcoxon rank test comparing the total population and recurrence population

P^b^ obtained from Wilcoxon rank test comparing the non-recurrence population and recurrence population

**Supplemental Table 3** Association between ratios of omega-3 PUFAs and EC recurrence^a^

| Variable | CI | HR | P-trend^b^ |
| --- | --- | --- | --- |
| EPA |  |  |  |
| Model 1 | (0.955,12.678) | 3.479 | 0.059 |
| Model 2 | (1.366,21.830) | 2.964 | **0.016** |
| DPA |  |  |  |
| Model 1 | (0.962,1.089) | 2.422 | 0.143 |
| Model 2 | (0.767, 11.463) | 2.964 | 0.115 |
| DHA |  |  |  |
| Model 1 | (0.967,1.098) | 2.328 | 0.168 |
| Model 2 | (0.671,8.242) | 2.352 | 0.181 |

^a^Cox regression was used to estimate the HRs and CIs. PUFAs were classified into dichotomies according to median

^b^P-trend values were conducted by assigning the median value to each tertile in the Cox regression models.

Model 1: adjusting for age

Model 2: adjusting for age, BMI, and stage, hormone therapy, and adjuvant therapy

**Supplemental Table 4** Omega-3 Fatty acid levels stratified by stage, grade, ER, and PR

|  | Total | Non-recurrence | recurrence | P^a^ | P^b^ |
| --- | --- | --- | --- | --- | --- |
| **stage-low** |  |  |  |  |  |
| C18:3n3 | 0.87（0.67-1.12) | 0.87（0.68-1.12) | 0.62（0.33-1.24) | 0.3173 | 0.3083 |
| C20:5n3 | 0.54（0.36-0.7) | 0.53（0.36-0.69) | 0.74（0.23-0.81) | 0.7174 | 0.7123 |
| C22:5n3 | 0.49（0.42-0.59) | 0.49（0.42-0.59) | 0.47（0.36-0.71) | 0.9853 | 0.985 |
| C22:6n3 | 2.61（2.21-3.1) | 2.6（2.21-3.1) | 2.77（2.59-3.37) | 0.4431 | 0.4346 |
| HUFA | 3.62（3.06-4.12) | 3.62（3.04-4.12) | 3.8（3.36-4.89) | 0.5683 | 0.561 |
| Total n-3PUFA | 4.54（3.92-5.22) | 4.53（3.92-5.22) | 5.04（3.98-5.22) | 0.6542 | 0.6482 |
| n-6 | 40.79（38.2-43.14) | 40.76（38.1-43.17) | 41.12（40.72-42.86) | 0.681 | 0.6754 |
| AA | 6.73（5.79-8.13) | 6.71（5.68-8.13) | 6.85（6.13-7.94) | 0.8204 | 0.8171 |
| AA/HUFA | 1.89（1.51-2.3) | 1.9（1.5-2.32) | 1.8（1.62-1.83) | 0.5683 | 0.561 |
| n-6/n-3 | 8.86（7.71-10.7) | 8.87（7.65-10.72) | 8.2（8.16-10.22) | 0.8684 | 0.866 |
| C20:5n3/ C18:3n3 | 0.56（0.39-0.82) | 0.56（0.39-0.82) | 0.6（0.37-2.44) | 0.672 | 0.6663 |
| C22:5n3/ C20:5n3 | 1.01（0.73-1.31) | 1.01（0.73-1.31) | 0.88（0.63-1.56) | 0.8109 | 0.8074 |
| **stage-high** |  |  |  |  |  |
| C18:3n3 | 0.75（0.63-0.93) | 0.7（0.6-0.8) | 0.93（0.92-1.19) | 0.0279 | 0.003 |
| C20:5n3 | 0.52（0.38-0.83) | 0.45（0.35-0.69) | 0.8（0.56-0.96) | 0.0955 | 0.0245 |
| C22:5n3 | 0.53（0.4-0.61) | 0.49（0.39-0.59) | 0.56（0.51-0.7) | 0.223 | 0.1001 |
| C22:6n3 | 2.73（2.23-3.25) | 2.59（2.21-3.16) | 2.87（2.4-3.39) | 0.6641 | 0.558 |
| HUFA | 3.63（3.1-4.48) | 3.53（3.01-4.39) | 4.25（3.31-4.71) | 0.4085 | 0.2649 |
| Total n-3PUFA | 4.43（3.95-5.17) | 4.25（3.79-4.98) | 4.97（4.31-5.99) | 0.1571 | 0.0563 |
| n-6 | 39.72（36.77-42.65) | 39.72（37-42.78) | 39.64（34.91-42.65) | 0.8997 | 0.8649 |
| AA | 7.37（6.48-8.04) | 7.37（6.67-8.04) | 7.31（5.98-8.31) | 0.7473 | 0.6638 |
| AA/HUFA | 2.01（1.59-2.38) | 2.03（1.65-2.4) | 1.92（1.35-2.19) | 0.3626 | 0.2193 |
| n-6/n-3 | 9.07（7.25-9.87) | 9.44（7.82-9.82) | 7.89（5.78-9.89) | 0.3626 | 0.2193 |
| C20:5n3/ C18:3n3 | 0.68（0.47-1.08) | 0.62（0.48-0.97) | 0.71（0.46-1.44) | 0.7053 | 0.6099 |
| C22:5n3/ C20:5n3 | 1（0.75-1.24) | 1.12（0.81-1.29) | 0.8（0.62-0.96) | 0.0955 | 0.0245 |
| **grade-low** |  |  |  |  |  |
| C18:3n3 | 0.86（0.67-1.06) | 0.84（0.67-1.04) | 1.06（0.77-1.29) | 0.2996 | 0.2763 |
| C20:5n3 | 0.54（0.36-0.7) | 0.51（0.36-0.67) | 0.79（0.65-0.89) | 0.0412 | 0.0321 |
| C22:5n3 | 0.49（0.42-0.6) | 0.49（0.42-0.59) | 0.56（0.49-0.68) | 0.1598 | 0.1401 |
| C22:6n3 | 2.62（2.21-3.11) | 2.61（2.21-3.1) | 2.83（2.18-3.38) | 0.6514 | 0.6354 |
| HUFA | 3.62（3.08-4.2) | 3.62（3.08-4.16) | 4.22（3.23-4.8) | 0.3917 | 0.3687 |
| Total n-3PUFA | 4.53（3.95-5.22) | 4.51（3.92-5.17) | 5.13（4.01-5.95) | 0.2288 | 0.2065 |
| n-6 | 41.1（38.1-42.98) | 41.1（38.2-43.1) | 40.92（35.28-41.81) | 0.3133 | 0.2899 |
| AA | 6.81（5.89-8.04) | 6.83（5.89-8.13) | 6.25（5.8-7.11) | 0.2896 | 0.2664 |
| AA/HUFA | 1.89（1.54-2.32) | 1.91（1.56-2.32) | 1.71（1.32-1.81) | 0.121 | 0.1037 |
| n-6/n-3 | 8.87（7.65-10.56) | 8.93（7.71-10.7) | 8.18（6.4-9.48) | 0.1895 | 0.1685 |
| C20:5n3/ C18:3n3 | 0.58（0.42-0.83) | 0.56（0.42-0.82) | 0.68（0.48-1.16) | 0.3274 | 0.304 |
| C22:5n3/ C20:5n3 | 1.01（0.73-1.3) | 1.01（0.73-1.31) | 0.76（0.63-1.02) | 0.1433 | 0.1245 |
| **grade-high** |  |  |  |  |  |
| C18:3n3 | 0.78（0.65-1.05) | 0.73（0.65-1.15) | 0.92（0.88-0.93) | 0.7631 | 0.7189 |
| C20:5n3 | 0.49（0.36-0.82) | 0.46（0.33-0.81) | 0.66（0.47-0.91) | 0.248 | 0.1677 |
| C22:5n3 | 0.5（0.45-0.59) | 0.48（0.42-0.57) | 0.52（0.45-0.7) | 0.5806 | 0.5094 |
| C22:6n3 | 2.56（2.2-2.98) | 2.41（2.09-3.07) | 2.84（2.78-2.89) | 0.1916 | 0.1189 |
| HUFA | 3.59（2.9-4.45) | 3.41（2.9-4.48) | 4.07（3.7-4.42) | 0.2691 | 0.1869 |
| Total n-3PUFA | 4.5（3.86-5.16) | 4.33（3.68-5.17) | 4.95（4.63-5) | 0.3659 | 0.2803 |
| n-6 | 38.91（37.01-43.17) | 38.69（36.87-43.14) | 42.65（37.86-46.36) | 0.4819 | 0.401 |
| AA | 7.23（5.84-8.53) | 6.91（5.7-8.55) | 8.31（7.63-8.52) | 0.1916 | 0.1189 |
| AA/HUFA | 1.92（1.52-2.41) | 1.76（1.51-2.46) | 2.06（2.04-2.19) | 0.6512 | 0.5893 |
| n-6/n-3 | 9.15（7.49-10.02) | 9.02（7.74-10.33) | 9.27（7.06-9.89) | 0.6512 | 0.5893 |
| C20:5n3/ C18:3n3 | 0.65（0.45-1.04) | 0.62（0.42-1.01) | 0.71（0.46-1.55) | 0.5467 | 0.4716 |
| C22:5n3/ C20:5n3 | 1.01（0.77-1.23) | 1.03（0.75-1.32) | 0.81（0.78-0.96) | 0.228 | 0.15 |
| **ER-negative** |  |  |  |  |  |
| C18:3n3 | 0.79(0.68-0.93) | 0.73(0.68-0.89) | 0.93(0.92-0.93) | 0.3082 | 0.2588 |
| C20:5n3 | 0.41(0.31-0.67) | 0.41(0.31-0.67) | 0.54(0.41-0.66) | 0.7644 | 0.7398 |
| C22:5n3 | 0.45(0.39-0.59) | 0.45(0.39-0.59) | 0.48(0.45-0.52) | 0.7644 | 0.7398 |
| C22:6n3 | 2.54(2.06-3.05) | 2.41(2.06-3.05) | 2.87(2.84-2.89) | 0.7644 | 0.7398 |
| HUFA | 3.24(2.9-4.53) | 3.16(2.9-4.53) | 3.89(3.7-4.07) | 0.5895 | 0.55 |
| Total n-3PUFA | 4.61(3.58-5.45) | 4.37(3.58-5.45) | 4.81(4.63-5) | 0.6748 | 0.642 |
| n-6 | 41.53(40.1-45.07) | 41.47(40.1-43.2) | 46.53(46.36-46.7) | 0.0479 | 0.0284 |
| AA | 7.36(5.45-8.31) | 6.81(5.45-8.17) | 7.97(7.63-8.31) | 0.3686 | 0.3191 |
| AA/HUFA | 1.89(1.21-2.4) | 1.62(1.21-2.4) | 2.05(2.04-2.06) | 0.5895 | 0.55 |
| n-6/n-3 | 9.88(6.84-11.84) | 9.88(6.84-11.84) | 9.68(9.27-10.1) | 0.9522 | 0.947 |
| C20:5n3/ C18:3n3 | 0.47(0.35-0.86) | 0.47(0.35-0.86) | 0.58(0.44-0.71) | 0.8573 | 0.8421 |
| C22:5n3/ C20:5n3 | 1.11(0.68-1.32) | 1.2(0.68-1.32) | 0.94(0.78-1.09) | 0.5895 | 0.55 |
| **ER-positive** |  |  |  |  |  |
| C18:3n3 | 0.84(0.65-1.08) | 0.83(0.65-1.06) | 0.97(0.88-1.24) | 0.2906 | 0.2547 |
| C20:5n3 | 0.55(0.36-0.74) | 0.54(0.35-0.72) | 0.79(0.56-0.96) | 0.0423 | 0.0286 |
| C22:5n3 | 0.52(0.43-0.61) | 0.52(0.42-0.6) | 0.56(0.47-0.7) | 0.2764 | 0.2407 |
| C22:6n3 | 2.62(2.26-3.14) | 2.62(2.25-3.12) | 2.83(2.4-3.39) | 0.5986 | 0.5704 |
| HUFA | 3.7(3.12-4.44) | 3.69(3.11-4.34) | 4.22(3.31-4.89) | 0.3732 | 0.3371 |
| Total n-3PUFA | 4.55(3.96-5.27) | 4.54(3.94-5.22) | 5.13(4.04-5.99) | 0.2304 | 0.1961 |
| n-6 | 41.1(37.32-43.02) | 41.1(37.32-43.1) | 40.92(35.88-42.19) | 0.3861 | 0.3501 |
| AA | 6.86(5.79-8.13) | 6.87(5.7-8.23) | 6.61(5.98-7.38) | 0.6097 | 0.5821 |
| AA/HUFA | 1.84(1.53-2.21) | 1.88(1.54-2.27) | 1.71(1.35-1.83) | 0.2068 | 0.1735 |
| n-6/n-3 | 8.74(7.53-10.22) | 8.82(7.63-10.39) | 8.18(5.78-9.89) | 0.2367 | 0.2021 |
| C20:5n3/ C18:3n3 | 0.6(0.44-0.86) | 0.59(0.43-0.86) | 0.68(0.46-1.44) | 0.2979 | 0.2618 |
| C22:5n3/ C20:5n3 | 0.97(0.73-1.31) | 1(0.75-1.33) | 0.76(0.62-0.96) | 0.0529 | 0.037 |
| **PR-negative** |  |  |  |  |  |
| C18:3n3 | 0.7(0.61-1.06) | 0.68(0.61-0.79) | 1.06(0.93-1.19) | 0.2915 | 0.2337 |
| C20:5n3 | 0.42(0.26-0.84) | 0.38(0.25-0.91) | 0.72(0.66-0.78) | 0.5268 | 0.4749 |
| C22:5n3 | 0.47(0.38-0.57) | 0.46(0.38-0.59) | 0.53(0.52-0.54) | 0.4392 | 0.3825 |
| C22:6n3 | 2.44(2.04-3.14) | 2.35(2.04-2.9) | 3.14(2.89-3.39) | 0.3607 | 0.302 |
| HUFA | 3.17(2.85-4.53) | 3.03(2.83-4.34) | 4.39(4.07-4.71) | 0.3607 | 0.302 |
| Total n-3PUFA | 4.57(3.42-5.48) | 4.08(3.42-5.05) | 5.45(5-5.9) | 0.3607 | 0.302 |
| n-6 | 40.43(38.61-42.23) | 40.25(37.88-41.47) | 43.89(41.43-46.36) | 0.2319 | 0.1771 |
| AA | 7.29(5.69-8.47) | 7.29(5.26-8.64) | 7.34(6.37-8.31) | 0.9439 | 0.9367 |
| AA/HUFA | 1.94(1.4-2.38) | 1.95(1.45-2.4) | 1.7(1.35-2.04) | 0.6226 | 0.5784 |
| n-6/n-3 | 9.3(7.25-11.68) | 9.82(7.49-11.84) | 8.14(7.02-9.27) | 0.5268 | 0.4749 |
| C20:5n3/ C18:3n3 | 0.51(0.34-1.16) | 0.47(0.33-1.48) | 0.68(0.65-0.71) | 0.6226 | 0.5784 |
| C22:5n3/ C20:5n3 | 1.12(0.71-1.38) | 1.22(0.73-1.44) | 0.74(0.7-0.78) | 0.3607 | 0.302 |
| **PR-positive** |  |  |  |  |  |
| C18:3n3 | 0.87(0.67-1.07) | 0.83(0.67-1.06) | 0.93(0.88-1.24) | 0.4226 | 0.3882 |
| C20:5n3 | 0.55(0.36-0.73) | 0.55(0.35-0.71) | 0.78(0.47-0.96) | 0.1029 | 0.0792 |
| C22:5n3 | 0.51(0.43-0.61) | 0.51(0.42-0.6) | 0.55(0.45-0.7) | 0.3426 | 0.3071 |
| C22:6n3 | 2.65(2.26-3.13) | 2.63(2.25-3.12) | 2.8(2.4-3.37) | 0.7952 | 0.78 |
| HUFA | 3.7(3.12-4.44) | 3.69(3.12-4.34) | 3.75(3.31-4.89) | 0.5691 | 0.54 |
| Total n-3PUFA | 4.55(3.97-5.28) | 4.55(3.95-5.27) | 4.83(4.04-5.99) | 0.3919 | 0.3569 |
| n-6 | 41.18(37.46-43.12) | 41.27(37.52-43.2) | 40.92(35.88-42.65) | 0.5586 | 0.529 |
| AA | 6.85(5.74-8.13) | 6.85(5.7-8.17) | 7.05(5.98-7.63) | 0.8555 | 0.8447 |
| AA/HUFA | 1.85(1.52-2.2) | 1.88(1.53-2.25) | 1.8(1.45-2.06) | 0.4881 | 0.4555 |
| n-6/n-3 | 8.84(7.58-10.24) | 8.86(7.63-10.39) | 8.47(5.78-10.1) | 0.5077 | 0.4759 |
| C20:5n3/ C18:3n3 | 0.59(0.44-0.86) | 0.59(0.44-0.86) | 0.65(0.44-1.44) | 0.4454 | 0.4115 |
| C22:5n3/ C20:5n3 | 1.00(0.73-1.3) | 1.005(0.75-1.31) | 0.84(0.62-1.09) | 0.1338 | 0.1067 |
|  |  |  |  |  |  |

Ⅰ-Ⅱ stage was defined as stage-low, more than Ⅱ stage was defined as stage-high; grade 1-2 was defined as grade-low, more than 2 was defined as grade-high

P^a^ obtained from Wilcoxon rank test comparing the total population and recurrence population

P^b^ obtained from Wilcoxon rank test comparing the non-recurrence population and recurrence population
